# Secretomics—A Key to a Comprehensive Picture of Unconventional Protein Secretion

**DOI:** 10.3389/fcell.2022.878027

**Published:** 2022-03-22

**Authors:** Gereon Poschmann, Jasmin Bahr, Jürgen Schrader, Ioana Stejerean-Todoran, Ivan Bogeski, Kai Stühler

**Affiliations:** ^1^ Institute for Molecular Medicine, Proteome Research, University Hospital and Medical Faculty, Heinrich-Heine-University Düsseldorf, Düsseldorf, Germany; ^2^ Department of Molecular Cardiology, University Hospital and Medical Faculty, Heinrich-Heine-University Düsseldorf, Düsseldorf, Germany; ^3^ Molecular Physiology, Institute for Cardiovascular Physiology, University Medical Center, Georg August University Göttingen, Göttingen, Germany; ^4^ Molecular Proteomics Laboratory, Biological Medical Research Center, Heinrich-Heine-University Düsseldorf, Düsseldorf, Germany

**Keywords:** Secretome, mass spectrometry, unconventional protein secretion, comparative secretomics, pharmacosecretomics, high-confident secretome (Min.5-Max. 8), proteomics

## Abstract

For a long time, leaderless secreted proteins (LLSP) were neglected as artifacts derived from dying cells. It is now generally accepted that secretion of LLSP–as a part of the collective term unconventional protein secretion (UPS) - is an evolutionarily conserved process and that these LLSP are actively and selectively secreted from living cells bypassing the classical endoplasmic reticulum-Golgi pathway. However, the mechanism of UPS pathways, as well as the number of LLSP and which part of a protein is involved in the selection of LLSPs for secretion, are still enigmatic and await clarification. Secretomics-a proteomics-based approach to identify and quantify all proteins secreted by a cell-is inherently unbiased toward a particular secretion pathway and offers the opportunity to shed light on the UPS. Here, we will evaluate and present recent results of proteomic workflows allowing to obtain high-confident secretome data. Additionally, we address that cell culture conditions largely affect the composition of the secretome. This has to be kept in mind to control cell culture induced artifacts and adaptation stress in serum free conditions. Evaluation of click chemistry for secretome analysis of cells under serum-containing conditions showed a significant change in the cellular proteome with longer incubation time upon treatment with non-canonical amino acid azidohomoalanine. Finally, we showed that the number of LLSP far exceeds the number of secreted proteins annotated in Uniprot and ProteinAtlas. Thus, secretomics in combination with sophisticated microbioanalytical and sample preparation methods is well suited to provide a comprehensive picture of UPS.

## Introduction

Secretomics - a proteomics-based approach to identify and quantify all proteins secreted by a cell - is inherently unbiased towards a specific secretion pathway and has been successfully applied in several research areas (for a detailed review, see (Mukherjee and Mani, 2013; Schaaij-Visser et al., 2013; Wei et al., 2021). However, sophisticated data analysis and experimental design allow meaningful conclusions about the underlying secretion pathways. To date, secreted proteins have been broadly classified into two classes. One class comprises secreted proteins that carry an N-terminal signal peptide that directs them to the ER-Golgi pathway. This secretion pathway, also known as “classical protein secretion,” is well proven and it had been shown that the signaling peptide hypothesis was both correct and universal because this process occurs in the same way in yeast, plant, and animal cells.

In contrast, the other classes of protein secretion, collectively termed “unconventional protein secretion” (UPS), are characterized by bypassing the ER-Golgi pathway and represents a group of proteins of unknown size. Currently, four types of pathways for UPS have been proposed (Rabouille, 2017; Dimou and Nickel, 2018). Three of them refer to soluble leaderless secreted proteins (LLSP) in the cytoplasm that are secreted either by 1) direct protein translocation through lipid pores in the plasma membrane (type I UPS), 2) plasma membrane-resident ABC transporters, with cargo proteins modified by acylation (type II UPS), or by uptake into endocytic compartments that mature and subsequently fuse with the plasma membrane (type III UPS). Type IV refers to plasma membrane proteins (with signal peptide) that are taken up into the ER but bypass the Golgi on their way to the cell surface. Detailed information is available only for a small group of LLSP that includes medically relevant proteins such as the cytokines FGF-1 and 2, IL-1a, IL-1b, IL-6, IL-18, and IL-33. To date, fibroblast growth factor-2 (FGF-2) is the best characterized candidate protein among these candidate proteins, exhibiting non-vesicular translocation through lipid-induced oligomerization and membrane insertion (Steringer et al., 2017).

The main reasons for the limited knowledge of UPS in contrast to classical protein secretion are that 1) UPS cannot be accurately predicted due to the still missing signaling patterns, 2) some UPS proteins also have an intracellular function (moonlighting), and therefore 3) the assignment of proteins to unconventional secretory pathways ultimately requires extensive experimental confirmation of extracellular function.

However, we believe that secretomics, in combination with rigorous experimental design and focused data analysis, is the key to a comprehensive picture of UPS. In this perspective, we will focus on recent advances and experimental settings in mass spectrometry (MS)-based secretomics that allow to shed light on the UPS. Here, we follow the definition that the secretome (as part of the conditioned medium) comprises bona fide secreted proteins whose abundance can be explained by experimental data (enrichment value), established knowledge of secretion, or predictions.

### High-Confident Secretome Data by Quantitative Secretomics

**FIGURE 1:**
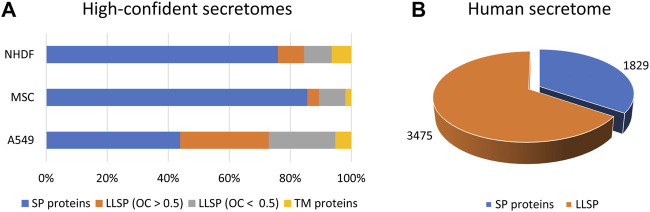
High-confident secretomes and the human secretome. **(A)** By means of comparative secretomics approach we were able to generate lists of high-confident secreted proteins of NHDF, MSC and A549 cells including 72–88% of proteins which were predicted to be secreted (Poschmann et al., 2021). **(B)** Our prediction tool OutCyte was used to estimate the number of candidates LLSP in the human secretome to 3,475 (Zhao et al., 2019). LLSP: leaderless secreted proteins. SP proteins: signal peptide containing proteins. OC: OutCyte score. TM proteins: transmembrane proteins.

Quantitative protein analysis by mass spectrometry is the method of choice in proteomics to characterize cellular compartments (Itzhak et al., 2017), interaction with proteins (Bensimon et al., 2012), nucleotides (Brillen et al., 2017), or drugs (Savitski et al., 2014), as this, in combination with specific fractionation steps, allows the determination of a significantly enriched protein population. We and others have shown that bona fide secreted proteins can be identified regardless of the secretion pathway when secretome and proteome data are compared by so-called “comparative secretomics” approach (Poschmann et al., 2021). Thus, we demonstrated that, depending on the cell line analyzed, comparative secretomics results in a high proportion of bona fide secreted proteins, with more than 30–70% being classically secreted proteins and 4–29% being candidate proteins released *via* unconventional secretion pathways (Figure 1A). Using this approach, more than 180 UPS candidate proteins have been identified, allowing the development of a novel prediction tool “OutCyte” [see below (Zhao et al., 2019)].

Quantitative secretomics has also been used to characterize leaderless secreted proteins through the so-called stable isotope dynamic labeling of secretomes (SIDLS) approach (Hammond et al., 2018). Dynamic isotope labeling of secretion kinetics can distinguish between secretory proteins and intracellular proteins released by cancer and stromal cells in culture. Interestingly, this study revealed a large number of LLSP with secretion kinetics comparable to classical secretory proteins, suggesting that the SIDLS approach is suitable for identifying continuously LLSP such as HDGF, PRDX2, AKR1B10, and C1QBP (Hammond et al., 2018).

Currently, we are working on combining quantitative secretomics and target identification by thermal proteome profiling in a so-called pharmacosecretomics approach. This approach aims to characterize the unconventional secretory pathways through a small molecule perturbation strategy. To date, there are only a limited number of small molecules with known targets that can be used for pharmacological modulation of UPS (Rodriguez-Furlan et al., 2017). Small molecule modulators that disrupt leaderless protein secretion allow functional dissection of components, their connectivity, and their regulators, especially within protein families (Rodriguez-Furlan et al., 2017). As suggested by Hick and Raikhe, this approach can be specific, robust, conditional, efficient, reversible, tunable, rapid, and simple (Hicks and Raikhel, 2012). In the first step, the change in secretory phenotype is determined by quantitative secretomics after treatment with small molecules with a previously unknown target spectrum. In the second step, the protein target involved in the secretory pathway is identified by thermal proteomic profiling (Savitski et al., 2014). Following this concept, new components and cargo associations of unconventional secretory pathways will become accessible.

In summary, quantitative secretomics provides the ability to identify LLSP either by their enrichment in the secretome or based on secretion kinetics. Thus, quantitative secretomics adds another level of quality control in addition to the simple bioinformatics filtering steps. However, these methods tend to be conservative, failing to detect LLSP with higher intracellular concentrations or slow secretion kinetics. In addition, quantitative secretomics combined with a small molecule perturbation strategy (pharmacosecretomics) has the potential to characterize secretory pathways and define subsets of LLSP that are preferentially secreted through specific pathways.

### Prediction of UPS and Current State of Secretome Data Bases

The development of prediction tools for UPS and thus the prediction of LLSP is more challenging than for classical protein secretion, because there are only a limited number of LLSP known and little is known about the different pathways involved. Currently, there are a number of prediction tools such as Outcyte, SecretomeP, SecretP, SPRED, or SRTpred, some of which differ in terms of sample set, taxa, algorithm, and prediction performance (for more details, see (Nielsen et al., 2019). For example, SecretomeP and SPRED use classical secretory proteins by removing their signal peptides based on the hypothesis that all secretory proteins share common features regardless of the specific secretory pathways. However, a recent benchmark has shown that SecretomeP performs much worse than originally thought, casting doubt on the underlying hypothesis. We therefore developed OutCyte, an integrated tool with two modules for predicting unconventional secretory proteins in eukaryotes (Zhao et al., 2019). In contrast to existing tools, the module for predicting potential UPS (OutCyte-UPS) was created with our in-house experimental secretome datasets using features generated directly from protein sequences. This allowed us to demonstrate that Outcyte outperforms SecretomeP and its successors, and we obtained information on important individual feature contributions for predictions using OutCyte. Among 61 tested physicochemical features, eight features were finally selected for tree boosting based machine learning. Among them, most important for the prediction of UPS were a higher frequency of arginine and other positively charged amino acids within the complete protein sequence as well as a relatively low molecular weight (∼21 kDa average MW of UPS candidates). Further features include the frequency of the aromatic amino acids tryptophan and phenylalanine as well as the frequency in the C-terminal 50 amino acids of: small amino acids, hydrophobic amino acids and polar amino acids. (Zhao et al., 2019).

Using Outcyte, we were able to roughly estimate the number of secreted proteins from the human proteome. Of 20,170 proteins, 1,829 were predicted to contain a signal for classical secretion (Figure 1B). This is consistent with other prediction tools/repositories: 1,836 proteins (SignalP 4.1), 1,693 proteins (DeepSig), and 1,999 proteins (UniProt). Surprisingly, we predicted 3,475 candidate LLSP proteins using Outcyte (Figure 1B). This far exceeds the number of the secretome annotated proteins (classically secreted and LLSP) in Uniprot (2,044 proteins) and ProteinAtlas (2,641 proteins) (Uhlen et al., 2019)).

The underrepresentation of LLSP in public available repositories interferes with comprehensive characterization extracellular space. For example, in an approach that aims to discover endocrine interactions by the integration of global multi-tissue expression and publicly available resources, LLSP might be underrepresented due to the incomplete annotation in UniProt KB (338 LLSP out of 2,248 proteins; 11%; Seldin et al., 2018). Nevertheless, this method, termed Quantitative Endocrine Network Interaction Estimation (QENIE), revealed an endocrine relationship between different tissues for seven LLSP (Xdh, Csn2, Nampt, Otop1, C1qbp, Ctf1, Fgf1), resulting in an overrepresentation of LLSP among the total number of candidate proteins (7 LLSP out of 47 proteins; 15%, *p* = 0.0499).

This example highlights both the biological role of LLSP and the need for dissemination and access to high-confidence secretome data through public repositories. In addition, the increasing amount of highly reliable secretome data and recent developments in machine learning are opening up new avenues that can not only allow to improve the prediction tools of unconventional protein secretion but also provide access to information on sequence motifs and cellular interactions.

### *In Vivo* and *in Vitro* Methods Allowing to Manage Secretion Artefacts

**FIGURE 2:**
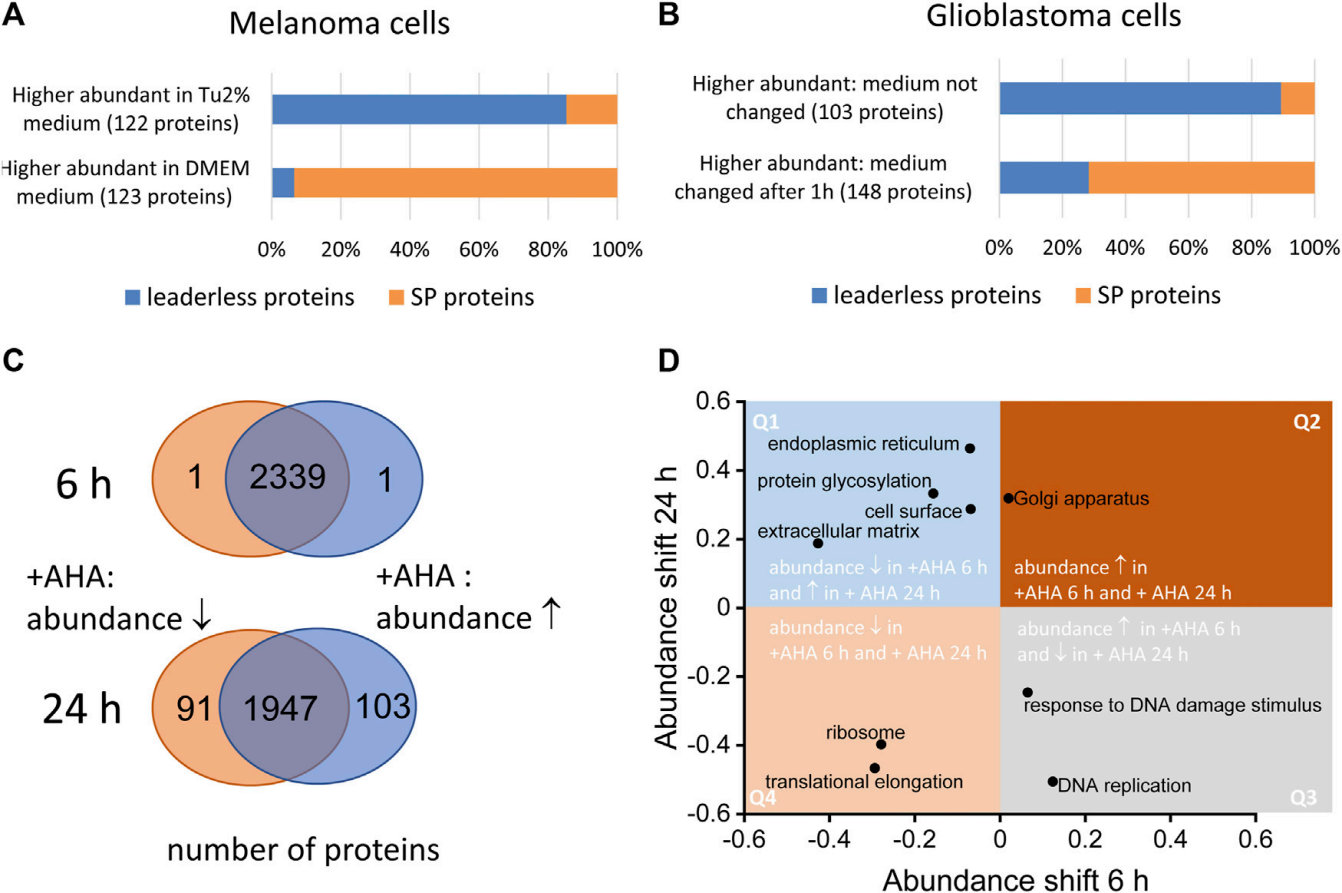
Effect of cell culture conditions on the secretome. Both serum-free and serum containing approached might influence the composition of secretomes. **(A)** WM3918 melanoma cells (n = 3 dishes per group) were cultivated for 24 in serum free medium. Cells expanded in DMEM showed a significantly higher proportion (*p*-value 2.2E-16, Fisher’s exact test) of signal-peptide containing proteins (SP proteins) at higher abundances in secretomes, whereas in Tu2% medium based secreteomes, putative LLSP showed higher abundances in comparison to expansion in DMEM. **(B)** LN18 glioblastoma cells (n = 3 per group) were incubated for 24 h in serum-free medium. After 1 h, the medium was replaced in one set of samples. In this samples, signal peptide containing proteins showed higher intensities in resulting secretome samples whereas in samples in which the medium was not changed after 1 h, a significant higher proportion of putative LLSP could be found at higher intensities (*p*-value 2.2E-16, Fisher’s exact test). **(C)** Normal human dermal foreskin fibroblasts were cultured with azidohomoalanine (AHA) or methionine as control for 6 and 24 h (n = 5 dishes per group). After MS analysis, different abundant proteins were determined by the Student’s *t*-test based significance analysis of microarrays approach (Tusher et al., 2001). Whereas after 6 h only 2 proteins (of 2441 cellular proteins) showed a significant AHA induced abundance change, 194 proteins (of 2141 cellular proteins) showed an abundance change after 24 h **(D)**. This dataset was also used for the analysis of global shifts in abundance on the level of proteins grouped by gene ontology annotations (Cox and Mann, 2012). Selected GOCC and GOBP categories are shown indicating the size of the abundance shift of associated proteins. Whereas proteins for some categories show an abundance change in the same direction after 6 and 24 h AHA incubation (found in quadrant Q2 and Q4), other protein groups show an AHA induced abundance shift in the opposite direction at the two timepoints (found in quadrant Q1 and Q3).

The main drawback in identifying of bona fide secreted proteins using secretomics from cultured cells are proteinaceous artefacts originating form cell culture medium, dying cells and artificial culture conditions that do not perfectly mimic the physiological environment. Although serum deprivation might impact cells’ viability, conditioned media obtained from serum-free cell cultivation is still the gold standard for secretomics. Nevertheless, in a number of studies, only limited information is available about detailed culture conditions and viability and changes of cells after serum deprivation. In this context, cell culture conditions should be optimized allowing a high viability of the cells without extensive amount of protein supplements (for more details we refer to (Schira-Heinen et al., 2019). It is important to note that the choice of medium can also have a major impact on the secretome of the cells. We found that, for example, WM3918 melanoma cells released a much higher proportion of signal peptide containing proteins and a lower proportion of LLSP in Dulbecco’s Modified Eagle Medium (DMEM) compared to expansion in Tu2% medium (Figure 2A). In secretomes from WM3918 cells expanded in the latter medium, we found the opposite: a large proportion of predicted LLSP and a comparable low number of signal peptide containing proteins. Tu2% medium is a mixture of 80% MCDB153 basal medium and 20% Leibovitz’s L-15 medium and contains 2% fetal calf serum and insulin whereas the used DMEM contained 10% fetal calf serum.

Furthermore, we revealed that renewal of serum-free medium after a short period (1 h) helps to avoid or measure potential stress artifacts originating from the adaptation to the serum-free medium conditions. In secretomes from LN18 cells cultured for 24 h under serum-free condition, the proportion of signal peptide containing proteins increased when the serum-free medium was exchanged after 1 h. When cells were incubated in serum-free medium for 24 h without exchanging the medium after 1 h, the proportion of putative LLSP in the secretome was significantly higher (Figure 2B).

However, in secretome approaches based on serum-free medium, contaminating proteins from serum that were not completely removed during the washing steps could remain in the secretome. Some groups use labeling of cells with heavy isotope labeled amino acids to deal with this issue (Polacek et al., 2010). Proteins which were only found with light amino acids included can be identified as serum contaminants in this approach. We found that -at least for cultures from human cells–it might also be possible to control contaminants from bovine serum just by tagging the respective proteins by contaminant lists as they were already included for example, in MaxQuant (Tyanova et al., 2016). In a study with normal human dermal foreskin fibroblasts which previously were labeled with heavy amino acids, we found 72 proteins with no intensity values in non-light channels which were all included in the contaminant list of MaxQuant and could therefore be removed without the need of additional isotope labeling.

As not every cell type can be cultured under serum deprivation without significant changes in cell viability, protein abundance or posttranslational modification (Hasan et al., 1999; Cooper, 2003), methods were developed to enable secretome preparation in serum-containing medium (Eichelbaum et al., 2012; Lai et al., 2013). For example, Eichelbaum and others used a two-dimensional metabolic labelling strategy based on pulsed stable isotope labelling of amino acids in cell culture (pSILAC) (Eichelbaum and Krijgsveld, 2014) and labelling with the biorthogonal, non-canonical amino acid azidohomoalanine (AHA) (Eichelbaum et al., 2012). The labeling strategy enables an enrichment of newly synthesized low abundant proteins by click chemistry on the azide-group of the AHA label as well as direct comparison of two different conditions by SILAC labeling. Although successfully applied caution have to be paid, when applying AHA labelling. We and others have shown that AHA-labelling changes the proteome of cells especially at longer incubation times (Figure 2C; (Eichelbaum et al., 2012)). Therefore, it is important to find a good compromise between longer incubation times, which might be necessary to collect a sufficient amount of secreted proteins for analysis and a potentially undesirable AHA-induced changes in cellular pathways. Those changes could already be detected after 6 h as for examples, ribosomal and translation associated proteins show a lower abundance in normal human dermal foreskin fibroblasts after incubation with AHA which is even more pronounced after 24 h (Figure 2D). Moreover, proteins associated with extracellular matrix, glycosylation and endoplasmic reticulum exhibit higher abundances in the cell upon 24 h AHA labeling and lower abundances after 6 h AHA labeling. This suggests that at least the classical secretion of proteins may be impaired during longer AHA incubation times, as we found related proteins predominately inside the cell at this time and in lesser amounts in the secretome.

Glycocapture, which is an additional tool to enrich secreted proteins under serum containing conditions (Lai et al., 2013) will probably more suited for secreted proteins of type IV UPS as glycosylation is a hallmark of secreted proteins facilitating the ER-Golgi route.

To make secretome analysis less susceptible to cell culture artifacts, both *ex vivo* and *in vivo* methods have been developed. For example, Roelofsen and others developed a method based on comparison of incorporation rates of isotope-labeled amino acids (CILAIR) to determine the secretome of human adipose tissue (Roelofsen et al., 2009). After incubation of human visceral adipose tissue from a patient in medium containing [^13^C]-lysine, 156 potentially secreted proteins were identified based on significant incorporation rates. Although this method allows the determination of secreted proteins from *ex vivo* tissue samples, it is biased toward proteins with rapid rates of protein synthesis and secretion and is therefore less suitable for proteins that are not continuously secreted. Although most LLSP are secreted under certain conditions such as stress, Hammond and others have shown that a large number of LLSP proteins also have a high secretion constant (Hammond et al., 2018). This suggests that the CILAIR approach is not only suitable for classical secreted proteins and allows LLSP to be monitored under *ex vivo* conditions from human tissue.

Recently, two publications used proximity biotinylation to characterize tissue-specific *in vivo* secretion in mice. Liu and others introduce the “secretome mouse,” a genetic platform that allows rapid identification of the cell- or tissue-specific *in vivo* secretome under basal conditions or after physiological or pathophysiological stress (Liu et al., 2021). Although they used an ER-BioID^HA^ construct containing the promiscuous biotinylation enzyme BioID2 in conjunction with the C-terminal ER retention sequence KDEL, they were confident in detecting LLSP of type III UPS in addition to classically secreted proteins because this pathway involves late endosomes, multivesicular bodies, or autophagosomes whose membrane is thought to be of ER origin. A similar approach that additionally targets UPS uses the Cyto-TurboID construct in combination with the Mem-TurboID and ER-TurboID constructs (Wei et al., 2021). After infection with the lentiviral constructs and feeding the mice with biotin, cell type-specific expression of the constructs was applied in mice to characterize nutrient-dependent reprogramming of the hepatocyte *in vivo* secretome. Thus, increased abundance of LLSP betaine homocysteine S-methyltransferase (BHMT) with a high cell type specific secretion was observed and functionally validated.

Although these approaches are unbiased toward a specific secretory pathway, they address a major pitfall in the field of UPS research, namely the avoidance of artifacts from *in vitro* cultivation.

## Concluding Remarks

As an omics technique, secretomics aims for the generation of a comprehensive picture of all proteins secreted by different cell types. Thus, secretomics will provide access to a vast array of LLSP which have been neglected so far. This will on the one hand enable to characterize the composition of the extracellular microenvironment leading to a deeper understanding of tissue homeostasis and cell-cell-communication and on the other hand give access to the elucidation of unconventional secretory pathways in respect of cargo selection and transport to the extracellular space. Regarding the latter aspect it will be interesting to follow how secretomics combined with machine learning will accelerate the elucidation of novel associations between cargo motifs and secretory pathway, as known for the IL-1b and motif-1 in TMED10-channeled UPS (Zhang et al., 2020) or the basic clusters of FGF2 in the lipid-induced oligomerization and membrane insertion associated UPS (Steringer et al., 2017).

## Data Availability

The datasets presented in this study can be found in online repositories. The names of the repository/repositories and accession number(s) can be found below: https://www.ebi.ac.uk/pride/archive/, PXD018895.
